# Long noncoding RNA AFAP1-AS1 promotes tumor progression and invasion by regulating the miR-2110/*Sp1* axis in triple-negative breast cancer

**DOI:** 10.1038/s41419-021-03917-z

**Published:** 2021-06-18

**Authors:** Xiaohui Zhang, Fangyuan Li, Yidong Zhou, Feng Mao, Yan Lin, Songjie Shen, Yuntao Li, Sheng Zhang, Qiang Sun

**Affiliations:** 1 Department of Breast Surgery, Peking Union Medical College Hospital, Peking Union Medical College &Chinese Academy of Medical Sciences (CAMS), Beijing, China; 2 Medical Science Research Centre, Peking Union Medical College Hospital, Peking Union Medical College &Chinese Academy of Medical Sciences (CAMS), Beijing, China; 3 No.1 department of surgery, the Fourth Hospital of Hebei Medical University, Shijiazhuang, China; 4 3rd Department of Breast Cancer, Tianjin Medical University Cancer Institute and Hospital, Tianjin, China

**Keywords:** Breast cancer, Long non-coding RNAs, Oncogenesis

## Abstract

Long noncoding ribonucleic acids (LncRNAs) have been found to be involved in the proliferation, apoptosis, invasion, migration, and other pathological processes of triple-negative breast cancer (TNBC). Expression of the lncRNA actin filament-associated protein 1 antisense RNA1 (AFAP1-AS1) has been found to be significantly higher in TNBC than in other subtypes or in normal tissue samples, but the specific mechanism by which AFAP1-AS1 affects the occurrence and development of TNBC is yet to be revealed. In this study, we used Cell Counting Kit-8 (CCK-8), colony formation, wound healing migration, Transwell invasion, and nude mouse xenograft assays to confirm the role of AFAP1-AS1 in the proliferation, migration of TNBC cells in vitro and in vivo. In addition, we performed bioinformatics analyses, reverse transcriptase quantitative polymerase chain reaction (RT-qPCR), western blot (WB), and dual-luciferase reporter assays (dual-LRA) to confirm interaction among AFAP1-AS1, micro-RNA 2110 (miR-2110), and *Sp1* transcription factor (*Sp1*). We found that silencing AFAP1-AS1 and *Sp1* or upregulating miR-2110 suppressed the proliferation, migration, and invasion of MDA–MB-231 and MDA–MB-468 cells in vitro as well as tumor growth in vivo. Mechanistically, the dual-LRA highlighted that miR-2110 was an inhibitory target of AFAP1-AS1, and that AFAP1-AS1 functioned as a miR-2110 sponge to increase *Sp1* expression. AFAP1-AS1 silencing led to a reduction in *Sp1* mRNA and protein levels, which could be reversed by joint transfection with miR-2110 inhibitor. Our findings demonstrated that AFAP1-AS1 could modulate the progression of breast cancer cells and affect tumorigenesis in mice by acting as a miR-2110 sponge, resulting in regulation of *Sp1* expression. Therefore, AFAP1-AS1 could play a pivotal role in the treatment of TNBC.

## Introduction

Breast cancer (BC) is one of the most common cancers among women worldwide. Data showed that BC accounted for 24.5% of newly diagnosed cancer cases and was the cause of 15.5% of cancer deaths in women during 2020 [1]. Clinically, according to the expression differences of estrogen receptor (ER), progesterone receptor (PR), and human epidermal growth factor receptor 2 (HER2), BC is divided into the following four types: Luminal A (ER^+^ and/or PR^+^, HER2^−^), Luminal B (ER^+^ and/or PR^+^, HER2^−/+^), HER2^+^ (ER^−^, PR^−^, HER2^+^) and triple-negative breast cancer (TNBC). Of these, the TNBC subtype accounts for about 15–20% of all BC cases, which is highly malignant and has the characteristics of high recurrence rate, high metastatic potential, poor treatment response and poor prognosis. There is no effective targeted therapy for TNBC other than conventional chemotherapy and radiotherapy. Therefore, it is urgent to further understand the molecular mechanism of TNBC tumor progression, and to develop experimental targets with potential clinical application, as well as to formulate more effective clinical treatment strategies and improve the prognoses of patients [2]. Of such experimental targets, RNA-based cancer treatment methods have gradually moved from concept to reality. Noncoding RNAs (ncRNAs) block mRNA function by inhibiting its transcription and binding to proteins, which exerts clinically therapeutic effects on tumors [3, 4].

Long noncoding RNAs (lncRNAs), which are longer than 200 nt, are one type of ncRNA; they have complicated biological functions and no protein-coding functions. Their abnormal expression or dysfunction is proven to be closely related to the occurrence and development of human diseases [5]. LncRNAs have been shown to be involved in proliferation, apoptosis, invasion, migration, epithelial-mesenchymal transition (EMT), and other pathological processes of TNBC. Based on the rapid development of high-throughput sequencing and the help of powerful bioinformatics analysis tools, a large amount of information on lncRNAs has emerged over the past decade. Reiche et al. identified >9500 lncRNA transcripts with significant expression differences between the normal breast tissue and cancer tissue [6]. A comprehensive analysis of lncRNAs expression profiles and clinical data from 1097 BC samples from The Cancer Genome Atlas (TCGA) showed that compared with normal samples, 1510 lncRNAs were differentially expressed in TNBC, and 672 lncRNAs differed in expression between TNBC and non-TNBC samples [7]. However, the specific mechanism by which lncRNAs affect the occurrence and development of TNBC has not been fully revealed. Therefore, exploring TNBC-related lncRNAs; studying the molecular mechanism of TNBC; and providing reference data for the understanding, diagnosis, treatment, and prognosis of TNBC are urgently needed.

The lncRNA actin fiber associated protein 1-antisense RNA1 (lncRNA–AFAP1-AS1, or AFAP1-AS1 for short) was first found in the sequencing of esophageal adenocarcinoma (EAC) and normal tissues. It was located on the anti-sense chain of the protein-coding gene AFAP1 on chr4: 7755817–7780655 (+) of human genome (GRCh37/hg19). AFAP1-AS1 is reported to be closely correlated with proliferation and metastasis of different cancers and is closely connected with poor prognosis of human malignancies [8], such as pancreatic ductal adenocarcinoma [9], EAC [10], lung cancer [11], and colorectal cancer [12].

In BC, significantly upregulated AFAP1-AS1 indicates poor prognosis [13, 14]. Analysis of seven pairs of HER2^+^ subtype BC samples has identified AFAP1-AS1 as the most dysregulated lncRNA in HER2^+^ subtype [15]. Limited knockout of AFAP1-AS1 (about 25–50%) is sufficient to reduce proliferation and colony formation of MDA–MB-231 and HCC1937 cells [16], meanwhile AFAP1-AS1 knockdown also represses BT-549 and MCF-7 cell proliferation and migration by downregulating septin-2 (*SEPT2*) via the sponging of miR-497-5p [17]. An in silico analysis of cDNA microarray data confirmed that AFAP1-AS1 is a potential prognostic factor in TNBC [18]. In addition, it promotes EMT via the *Wnt*/*β-catenin* signaling pathway in TNBC [19]. A previous study in our laboratory analyzed AFAP1-AS1 in BC samples from TCGA and found that its expression level in TNBC was significantly higher than in other subtypes or in normal tissue samples [20]. Taken together, the aforementioned results suggest that AFAP1-AS1 is involved in the pathogenesis of TNBC and could become a new biomarker or therapeutic target in the treatment thereof. However, despite a limited amount of research, our understanding of the roles that AFAP1-AS1 specific targets and related signaling pathways play in the development and progression of TNBC has remained fragmentary and needs further study.

In this study, bioinformatics analysis, molecular biology, cell biology, and a tumorigenesis assay in nude mice were used to study the downstream regulatory relationships of AFAP1-AS1. We found that AFAP1-AS1 and *Sp1* transcription factor (*Sp1*) knockdown or miR-2110 overexpression suppressed MDA–MB-231 and MDA–MB-468 cell proliferation, migration, and invasion in vitro as well as tumor growth in vivo. AFAP1-AS1 silencing led to a reduction in *Sp1* mRNA and protein levels, which could be reversed by joint transfection with miR-2110 inhibitor. Mechanistically, the results revealed that AFAP1-AS1 competitively bound to miR-2110, affecting the expression of *Sp1*, and thereby regulating proliferation and migration of TNBC cells and tumor progression in vivo.

## Results

### AFAP1-AS1 expression was upregulated in human TNBC cells and tissues

Reverse transcriptase quantitative polymerase chain reaction (RT-qPCR) results showed that AFAP1-AS1 was significantly upregulated in three TNBC cell lines (BT-549, MDA–MB-231, and MDA–MB-468) compared with the normal human breast epithelial cell line MCF-10A (Fig. S1A).

In addition, we verified the level of AFAP1-AS1 expression in eight pairs of TNBC samples and in histologically normal tissues using RT-qPCR, with AFAP1-AS1 normalized to glyceraldehyde-3-phosphate dehydrogenase (*GAPDH*; Table S2). AFAP1-AS1 expression was significantly upregulated in cancerous tissues (mean ratio, 3.02-fold; *P* < 0.05) compared with their normal counterparts (Fig. S1D).

Examining the correlation between AFAP1-AS1 expression and clinicopathological features showed that AFAP1-AS1 upregulation was correlated with tumor size (*P* = 0.0279; Fig. S1G). However, it was found not associated with lymph node metastasis (Fig. S1H, I). These results implied that AFAP1-AS1 overexpression might be useful in the development of novel progression markers for TNBC.

### AFAP-AS1 promoted the proliferation, migration, and invasion of TNBC cells

**Fig. 1:**
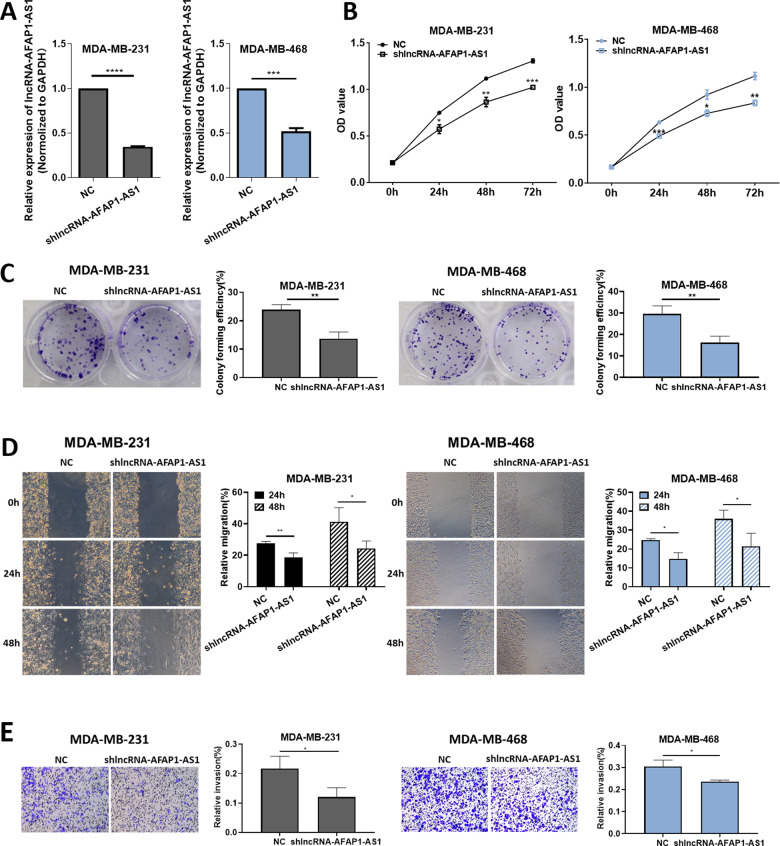
AFAP1-AS1 promoted the proliferation, migration, and invasion of TNBC cells. **A** Relative expression of AFAP1-AS1 after treatment with shlncRNA–AFAP1-AS1. **B** CCK-8 assay results showed the effect of shlncRNA–AFAP1-AS1 on the proliferation of MDA–MB-231 and MDA–MB-468 cells at 0, 24, 48, and 72 h after transfection. **C** Colony formation, **D** wound healing migration and **E** Transwell invasion assays were performed on MDA–MB-231 and MDA–MB-468 cells. Unpaired student’s *t*-test and one-way ANOVA test were used for the statistical analyses. *P < 0.05; **P < 0.01; ***P < 0.005;****P < 0.001; ns not significant.

To explore the biological function of AFAP1-AS1, first we designed a short-hairpin RNA (shRNA), shlncRNA–AFAP1-AS1. Compared with the negative control (NC) group, expression of AFAP1-AS1 was significantly decreased in MDA–MB-231 and MDA–MB-468 cells, as confirmed by RT-qPCR (Fig. 1A). CCK-8 results showed that cell viability was inhibited after the downregulation of AFAP1-AS1 (Fig. 1B), while colony formation assay results indicated that cell proliferation was decreased after treatment with shlncRNA–AFAP1-AS1 (Fig. 1C). Wound healing migration and Transwell invasion assays revealed that AFAP1-AS1 knockdown decreased cell migration and invasion, suggesting that AFAP1-AS1 was positively correlated with cell migration and invasion (Fig. 1D, E).

### AFAP1-AS1 targeted miR‑2110 in TNBC cells

An increasing number of studies have shown that lncRNAs might act as sponges of miRNA, thereby interfering with tumor progression. Therefore, we speculated that AFAP1-AS1 might influence the function of certain miRNAs that might play particular roles in TNBC.

**Fig. 2:**
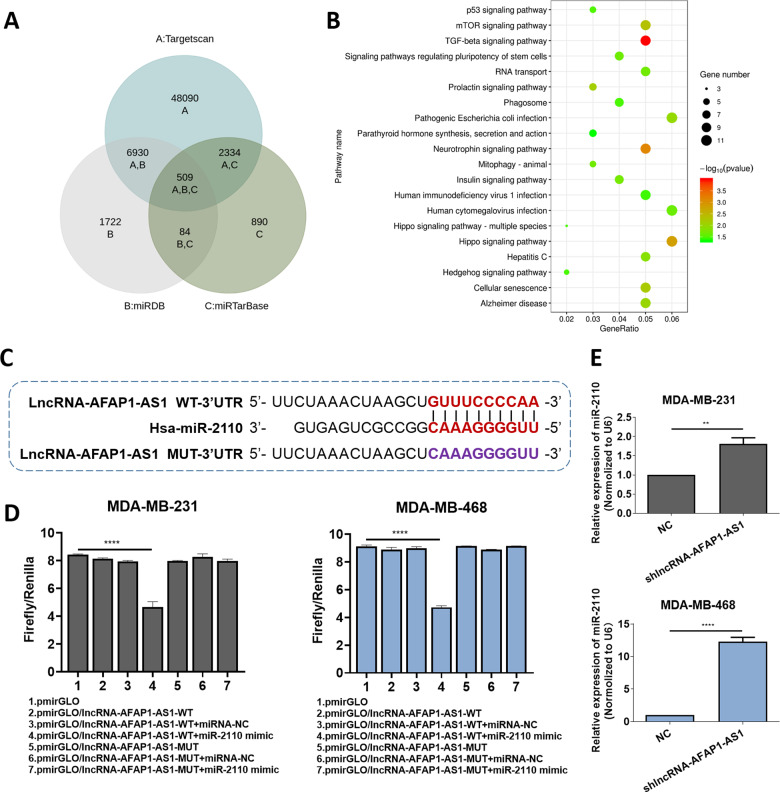
AFAP1-AS1 targeted miR‑2110 in TNBC cells. **A** Schematic illustration showing the overlap among 13 miRNA target mRNAs in three databases. **B** KEGG analysis of 462 predicted target genes. **C** Putative binding site of miR-2110 with AFAP1-AS1 3′ UTR. **D** The luciferase activity of pmirGLO/lncRNA–AFAP1-AS1-WT/MUT plasmid in MDA–MB-231 and MDA–MB-468 cells after co-transfection with miR-2110. **E** Relative miR-2110 expression with shlncRNA–AFAP1-AS1 or NC. Unpaired student’s *t*-test and one-way ANOVA test were used for the statistical analyses. *P < 0.05; **P < 0.01; ***P < 0.005; ****P < 0.001; ns not significant.

In a bioinformatics prediction assay, the LncBase database predicted 13 potential AFAP1-AS1 target miRNAs with scores ≥0.9 (Table 1). After using three miRNA interaction—TargetScan (http://www.targetscan.org/), miRDB (http://mirdb.org/) and miRTarBase (https://bio.tools/mirtarbase)—to predict miRNAs-mRNA interaction, we found 509 potential target miRNA-mRNA pairs for 13 miRNAs (Table 1). Next, we analyzed all the 462 unduplicated genes in the 509 pairs using R cluster profiler package. Combined with subsequent Kyoto Encyclopedia of Genes and Genomes (KEGG) analysis and review of studies on tumors that report specific pro-oncogenic and upregulated genes, indicated that the transforming growth factor beta (*TGF-β*) signaling pathway was enriched with 10 genes: *Sp1*, activin A receptor type 1B (*ACVR1B*), Smad family member 5 (*SMAD5*), neuroblastoma 1 (*NBL1*), nodal growth differentiation factor (*NODAL*), bone morphogenetic proteins 8A and 8B (*BMP8A*, *BMP8B*), *Smad*-specific E3 ubiquitin-protein ligase 2 (*SMURF2*), mitochondrial inner membrane organizing system 1-neuroblastoma 1 (*MINOS1*-*NBL1*) and mitogen-activated protein kinase (*MAPK1*; Fig. 2B). Four of these (*Sp1*, *NBL1*, *MINOS1*-*NBL1*, and *MAPK1*) were predicted to be target genes of miR-2110; hence, we selected miR-2110 according to its probable cancer-related downstream pathway.

**Table 1:** Search for miRNAs that could have potential interactions with AFAP1-AS1 (ENSG00000272620) via LncBase database.

transcript	lncRNA	miRNA	Score
ENST00000608442	ENSG00000272620(AFAP1-AS1)	hsa-miR-4695-5p	0.9950371666
ENST00000608442	ENSG00000272620(AFAP1-AS1)	hsa-miR-877-3p	0.9663345859
ENST00000608442	ENSG00000272620(AFAP1-AS1)	hsa-miR-9500	0.9656390921
ENST00000608442	ENSG00000272620(AFAP1-AS1)	hsa-miR-3612	0.942308032
ENST00000608442	ENSG00000272620(AFAP1-AS1)	hsa-miR-650	0.937535736
ENST00000608442	ENSG00000272620(AFAP1-AS1)	hsa-miR-4731-5p	0.9224101624
ENST00000608442	ENSG00000272620(AFAP1-AS1)	hsa-miR-5008-5p	0.9200537189
ENST00000608442	ENSG00000272620(AFAP1-AS1)	hsa-miR-7160-5p	0.9192748355
ENST00000608442	ENSG00000272620(AFAP1-AS1)	hsa-miR-431-5p	0.9163180819
ENST00000608442	ENSG00000272620(AFAP1-AS1)	hsa-miR-3144-5p	0.9149172503
ENST00000608442	ENSG00000272620(AFAP1-AS1)	hsa-miR-5699-3p	0.9134572776
ENST00000608442	ENSG00000272620(AFAP1-AS1)	hsa-miR-4779	0.9123530317
**ENST00000608442**	**ENSG00000272620(AFAP1-AS1)**	**hsa-miR-2110**	**0.9041805157**

Bold values indicate that miR-2110 was selected for the subsequent research.

MiR-2110 expression was dysregulated in human TNBC cells and tissues. RT-qPCR results showed that miR-2110 was decreased in TNBC cell lines BT-549, MDA–MB-231, and MDA–MB-468, compared with MCF-10A (Fig. S1B). The same trend appeared in TNBC tissues (Fig. S1E), indicating that miR-2110 expression was negatively correlated with AFAP1-AS1. Double luciferase reporter assays (dual-LRAs) were carried out via transfecting MDA–MB-231 and MDA–MB-468 cells with luciferase reporter vectors (containing wild-type or mutant sequences of miRNA targets). Compared with that of mutant plasmid (pmirGLO/lncRNA–AFAP1-AS1-MUT), luciferase reporter activity was significantly decreased by miR-2110 mimics in cells transfected with wild-type plasmid (pmirGLO/lncRNA–AFAP1-AS1-WT; Fig. 2D). Meanwhile, after AFAP1-AS1 was silenced, miR-2110 levels were upregulated compared with the NC group (Fig. 2E). Taken together, our results provided evidence that miR-2110 was negatively correlated with AFAP1-AS1 expression, and was the direct target of AFAP1-AS1 in TNBC cells.

### AFAP1-AS1 upregulated *Sp1* levels via sponging miR-2110

According to previous studies, miR-2110 has differentiation-inducing and oncosuppressive functions in neuroblastoma, and is significantly correlated with patient survival rate [21]. Therefore, we hypothesized that miR-2110 might function as a suppressor in TNBC cells and that AFAP1-AS1 promoted tumor progression by protecting downstream oncogenes from downregulation by miR-2110.

**Fig. 3:**
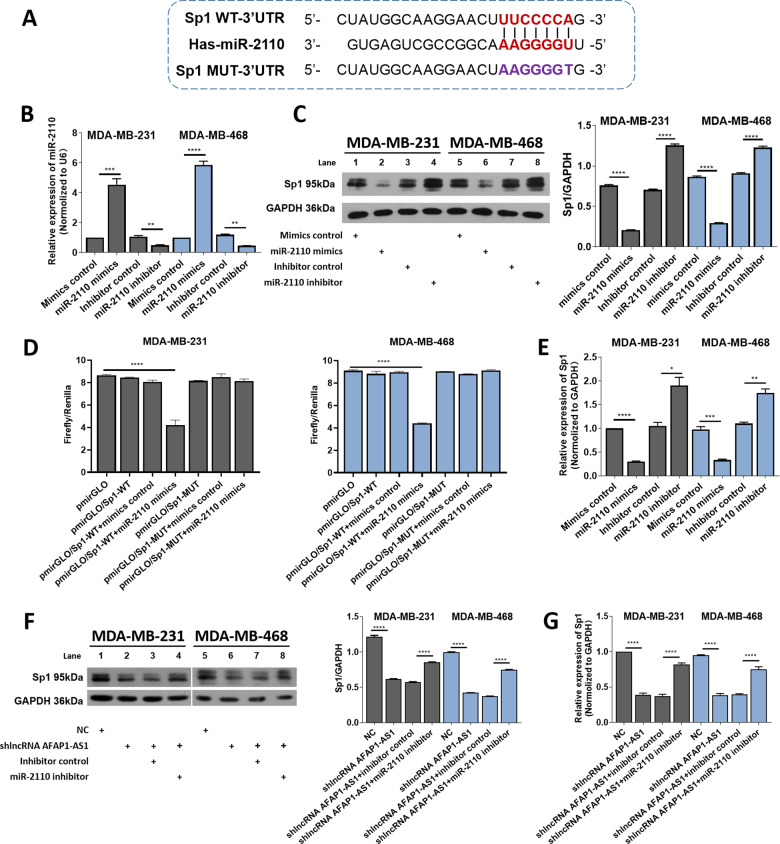
AFAP1-AS1 upregulated *Sp1* level via sponging miR-2110. **A** Putative binding site of miR-2110 with *Sp1* 3′ UTR. **B** MiR-2110 expression in MDA–MB-231 and MDA–MB-468 cells were examined through RT-qPCR. **C** Relative protein level of *Sp1* after miR-2110 mimics or inhibitor treatment. **D** The luciferase activity of pmirGLO/*Sp1*-WT/MUT plasmid after co-transfection with miR-2110. **E** The relative mRNA level of *Sp1* after miR-2110 mimics or inhibitor treatment. **F**, **G** Relative protein and mRNA level of *Sp1* after shlncRNA–AFAP1-AS1, miR-2110 mimics, and inhibitor treatment. Unpaired student’s *t*-test and one-way ANOVA test were used for the statistical analyses. *P < 0.05; **P < 0.01; ***P < 0.005; ****P < 0.001; ns not significant.

We screened five downstream genes with the highest correlations with miR-2110 (Table 2) and found that miR-2110 possessed binding sites in the 3ʹ untranslated regions (3ʹ UTR) of the predicted genes (Fig. 3A). First, miR-2110 mimics or inhibitors were transfected into MDA–MB-231 and MDA–MB-468 cells to observe changes in protein expression levels of candidate target genes (Fig. 3B). The results showed that compared with claudin 4 (*CLDN4*) [22, 23], RALY RNA binding protein like (*RALYL*) [24], rhomboid domain containing 1 (*RHBDD1*) [25, 26], and zinc finger protein 703 (*ZNF703*) [27], protein level expression of *Sp1* decreased significantly after transfection with miR-2110 mimics, while the expression of *Sp1* increased after transfection with miR-2110 inhibitor (Fig. 3C, S2). MiR-2110 is speculated to have the strongest ability to target *Sp1* [28–30], therefore, we subsequently conducted a dual-LRA to verify the target relationship between miR-2110 and *Sp1*. The results in MDA–MB-231 and MDA–MB-468 cells showed that relative luciferase reporter intensity was 50% lower after transfection with miR-2110 mimics compared with wild-type plasmid alone (pmirGLO/*Sp1*-WT), while miR-2110 mimics had no effect on mutation plasmid (pmirGLO/*Sp1*-MUT), which demonstrated that *Sp1* was a critical target of miR-2110 (Fig. 3D).

**Table 2:** Predicted target genes of miR-2110.

miRNA	Potential targets	Function in BC
hsa-miR-2110	Sp1	Associated with poor prognosis [28]; promotion of migration and invasion in BC cells [29, 30].
	CLDN4	Promoting BC cell proliferation, migration [22]; correlated positively with tumor grade and Her2, and negatively with ER [23].
	RALY	Increasing expression in BC cells and tumors, and correlated with decreased patient survival [24].
	RHBDD1	Promoting BC progression [25] and metastasis [26].
	ZNF703	BC oncogene, overexpressed in BC [27].

To further demonstrate the link among AFAP1-AS1, miR-2110, and *Sp1* expression, we subjected cells to different transfections. First, RT-qPCR showed that *Sp1* mRNA levels were significantly decreased after transfected with miR-2110 mimics in MDA–MB-231 and MDA–MB-468 cells, meanwhile the miR-2110 inhibitor caused an upregulated mRNA level of *Sp1* (Fig. 3E). Moreover, we transfected cells with NC or shlncRNA–AFAP1-AS1 and found that AFAP1-AS1 silencing led to a reduction in *Sp1* mRNA and protein levels, which could be rescued by the co-transfection of miR-2110 inhibitor (Fig. 3F, G). *Sp1* expression was then found to be dysregulated in human TNBC cells and tissues. RT-qPCR results showed that *Sp1* was overexpressed in TNBC cell lines (BT-549, MDA–MB-231, and MDA–MB-468) compared with MCF-10A cells (Fig. S1C). The same trend appeared in TNBC tissues (Fig. S1F), indicating that *Sp1* was positively correlated with AFAP1-AS1 expression. Taken together, our results showed that *Sp1* expression levels were negatively correlated with miR-2110, but positively correlated with AFAP1-AS1, suggesting that AFAP1-AS1 upregulated *Sp1* expression via sponging miR-2110.

Next, we performed Western blot (WB) experiments to show the upstream and downstream protein effects of AFAP1-AS1 on *Sp1* signaling. We verified the expressions of *Sp1* upstream proteins phosphorylated p38 (*p-p38*) and phosphorylated C-jun N-terminal kinase (*p-JNK*) and *Sp1* downstream proteins human homolog of mouse double minute 2 (*MDM2*), vascular endothelial growth factor (*VEGF*) and *survivin* under AFAP1-AS1 inhibition. Results showed that the reduction in AFAP1-AS1 did not affect protein expression of *p-p38* or *p-JNK*, but it modulated that of *survivin*, *VEGF*, and *MDM2* (Fig. S3), indicating that the downstream pathway of *Sp1* was regulated by AFAP1-AS1. However, there were no effects on the upstream *MAPK* signaling pathway.

### The AFAP1-AS1/miR-2110/*Sp1* axis affected the proliferation, migration, and invasion of TNBC cells

**Fig. 4:**
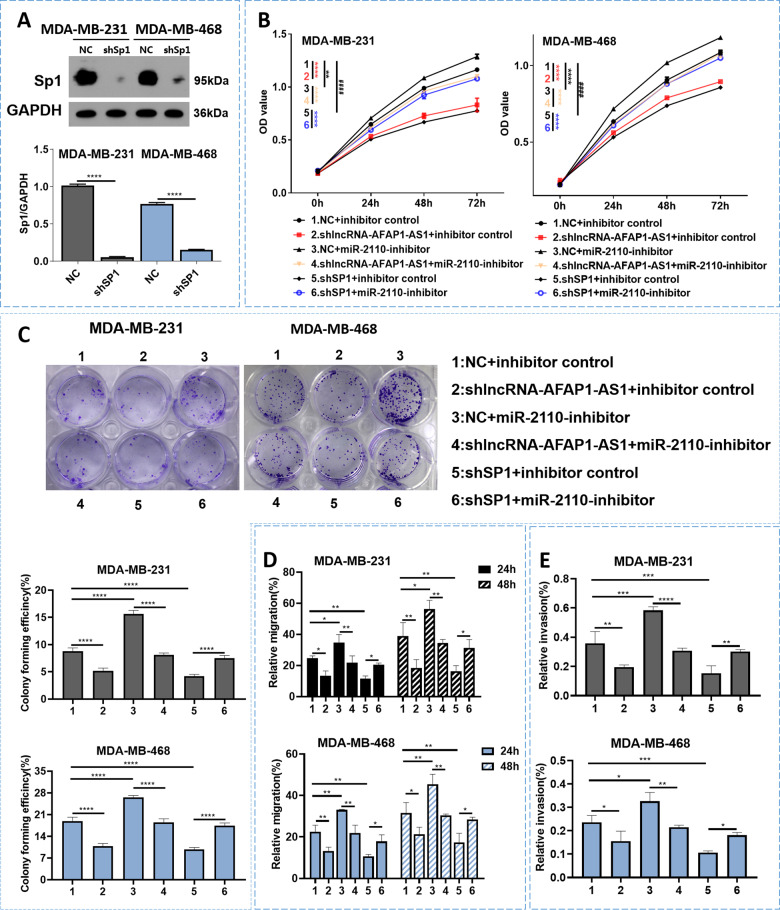
AFAP1-AS1/miR-2110/*Sp1* affected the proliferation, migration, and invasion of TNBC cells. **A** *Sp1* expression in MDA–MB-231 and MDA–MB-468 cells were examined through WB. **B** CCK-8 assay performed. Asterisks (*) and hash signs (**#**) represent the significant analysis between group 1 and group 2 (*), group 1 and group 3 (*), group 3 and group 4 (*), group 1 and group 5 (**#**), group 5 and group 6 (*), respectively. **C** Colony formation, **D** wound healing migration, and **E** Transwell assays performed after different treatment. Unpaired student’s *t*-test and one-way ANOVA test were used for the statistical analyses. *P < 0.05; **P < 0.01; ***P < 0.005; **** *P* < 0.001; ns not significant.

To further verify whether AFAP1-AS1 induced TNBC progression via miR-2110 sponging, we performed rescue experiments involving CCK-8, colony formation, wound healing, and Transwell invasion assays. The results showed that miR-2110 inhibition promoted cell proliferation, migration, and invasion (Fig. 4B–E; group 1 vs. group 3), meanwhile these phenotypes were all dismissed by AFAP1-AS1 silencing via co-transfection of shlncRNA–AFAP1-AS1 (Fig. 4B–E; group 3 vs. group 4). These results showed that AFAP1-AS1 silencing reversed the miR-2110 downregulation-induced phenotype of TNBC cells.

We then evaluated the roles of *Sp1* in TNBC cells via *Sp1* knockdown. After transfection, we verified *Sp1* expression using WB and found that it was significantly decreased in the sh*Sp1* group compared with the NC group (Fig. 4A). CCK-8 and colony formation assays showed that cell viability was inhibited after *Sp1* knockdown, while wound healing migration and Transwell invasion assays revealed that *Sp1* knockdown decreased migration, invasion of TNBC cells (Fig. 4B–E; group 1 vs. group 5 and Fig. S4). All of these effects were offset by the joint addition of miR-2110 inhibitor (Fig. 4B–E; group 5 vs. group 6 and Fig. S4). Taken together, these results indicated that downregulated *Sp1* inhibited tumor progression and that miR-2110 downregulation reversed the *Sp1* silencing-induced phenotype of TNBC cells.

### The AFAP1-AS1/miR-2110/*Sp1* axis affected tumorigenesis in mice

Mice were subcutaneously injected with MDA–MB-231 or MDA–MB-468 cells according to the following groupings: 1.NC + inhibitor control; 2.shlncRNA–AFAP1-AS1 + inhibitor control; 3.NC + miR-2110-inhibitor; 4.shlncRNA–AFAP1-AS1 + miR-2110-inhibitor; 5.sh*Sp1* + inhibitor control; 6.sh*Sp1* + miR-2110-inhibitor. We used these groups to investigate the role of the AFAP1-AS1/miR-2110/*Sp1* axis in tumor formation.

**Fig. 5:**
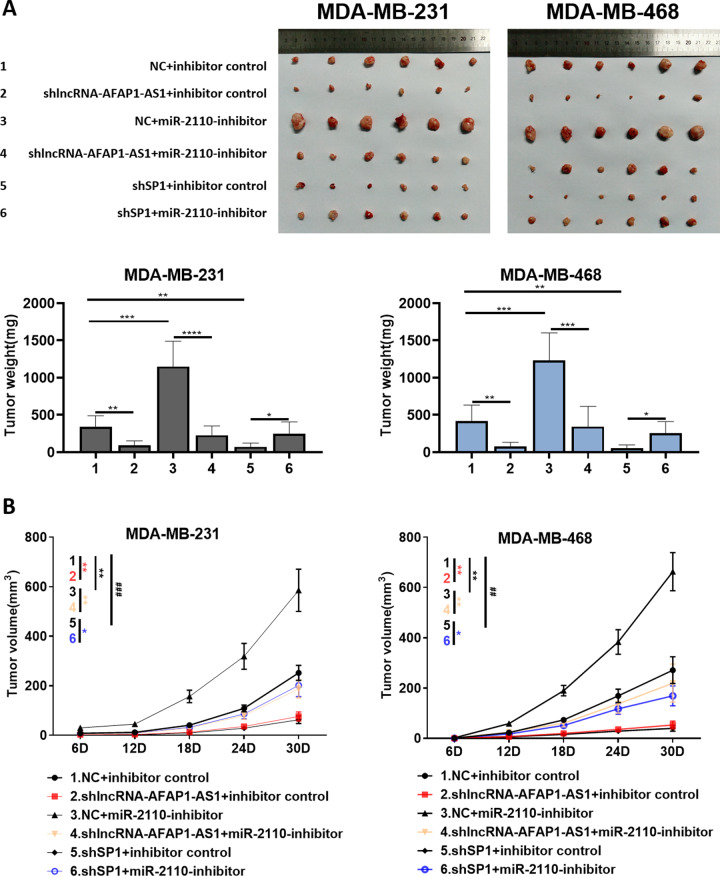
AFAP1-AS1/miR-2110/*Sp1* affected the tumorigenesis in mice. **A** Tumor tissues, tumor weight, and **B** growth curve in each group are shown. Asterisks (*) and hash signs (**#**) represent the significant analysis between group 1 and group 2 (*), group 1 and group 3 (*), group 3 and group 4 (*), group 1 and group 5 (**#**), group 5 and group 6 (*), respectively. Unpaired student’s *t*-test and one-way ANOVA test were used for the statistical analyses. *P < 0.05; **P < 0.01; ***P < 0.005; ****P < 0.001; ns not significant.

The findings showed that tumors in the AFAP1-AS1-silenced group had a slower growth rate and, in particular, less average volume and weight (Fig. 5; group 1 vs. group 2). In addition, tumor volume and weight in the miR-2110-silenced group increased significantly (Fig. 5; group 1 vs. group 3). These phenotypes were all reversed by AFAP1-AS1 silencing in the shlncRNA–AFAP1-AS1 + miR-2110-inhibitor group (Fig. 5; group 3 vs. group 4), which did not significantly differ from the NC group in tumor size and growth rate (Fig. 5; group 1 vs. group 4). At the same time, the silencing of *Sp1* also caused a significant decrease in tumor volume and growth rate (Fig. 5; group 1 vs. group 5). After the joint addition of miR-2110 inhibitor (sh*Sp1* + miR-2110-inhibitor group), related parameters such as tumor volume and growth rate increased (Fig. 5; group 5 vs. group 6). Then, we verified protein levels of *Sp1* in mouse tumors, which showed similar change trends in tumor size and growth rate (Fig. S5). This indicated that AFAP1-AS1 silencing led to a reduction in *Sp1* protein levels in mouse tumor. Taken together, these results showed that the AFAP1-AS1/miR-2110/*Sp1* axis affected the growth rate, average size, and weight of tumors.

## Discussion

A large number of lncRNAs have recently been discovered in functional genomics studies. Although many studies have shown that lncRNAs can participate in the pathological process of BC, relatively few on TNBC-related lncRNAs exist, and, in particular, the specific mechanism by which these lncRNAs affect the occurrence and development of TNBC has not yet been revealed in full.

AFAP1-AS1 was found on the anti-sense chain of the protein-coding gene *AFAP1* and is involved in the development of a variety of cancers. In gastric cancer tissues and cells, it is significantly upregulated and it regulates the proliferation and apoptosis of gastric cancer cells through the phosphatase and tensin homolog/phosphorylated protein kinase B (*PTEN*/*p-Akt*) pathway [31]. The expression of AFAP1-AS1 is also upregulated in esophageal squamous cell carcinoma, and is significantly correlated with tumor, node, and metastasis (TNM) stage and tumor size [32]. Hypo-methylation and high expression of AFAP1-AS1 have been found in Barrett’s esophagus and EAC; interference with AFAP1-AS1 expression can inhibit the proliferation and colony-forming ability of EAC cells [33]. AFAP1-AS1 is also significantly upregulated in BC tissues compared with normal tissues [13]. In our previous study, after analyzing the expression of AFAP1-AS1 in BC samples from the TCGA database, we found that expression of AFAP1-AS1 in TNBC was significantly higher than in other subtypes and in normal tissue sample [20]. Herein, we found AFAP1-AS1 to be overexpressed in TNBC tissues compared with normal tissues, suggesting that AFAP1-AS1 might be involved in the pathogenesis of TNBC and could become a new biomarker or therapeutic target. After discovering the potential importance of AFAP1-AS1, we studied its function on TNBC cells in depth and found that it could promote cell proliferation and clone formation and was positively correlated with cell migration and invasion. Our research will undoubtedly enrich the study of lncRNAs in TNBC.

Other studies have shown that lncRNAs participate in multiple regulatory mechanisms, such as regulating downstream mRNAs as competing endogenous RNAs (ceRNAs), as well as regulation of transcription, translation, protein modification, and formation of RNA-protein or protein-protein complexes. The ceRNA networks link the functions of mRNAs with miRNAs and lncRNAs, which leads us to assume that lncRNAs serve as miRNA sponges to eliminate miRNA of target genes. In this study, a dual-LRA confirmed that AFAP1-AS1 acted as a sponge for miR-2110, which was inversely correlated with AFAP1-AS1 expression. There are few studies on miR-2110, but it is considered a tumor suppressor in neuroblastoma [21] that might functioned by directly targeting *Tsukushi* [34]. Meanwhile, our research also proved that a decrease in miR-2110 promoted proliferation, migration, and invasion by TNBC cells. MiRNAs are capable of regulating physiological processes by inhibiting target mRNA translation or promoting mRNA degradation. The bioinformatics results of miRNA target prediction showed that the following five genes had the highest correlations with miR-2110 and could be potential targets thereof: *CLDN4*, *RALYL*, *RHBDD1*, *ZNF703*, and *Sp1*. Compared with that of the other four genes, *Sp1* expression was significantly more distinct after miR-2110 mimics and inhibitor transfection. *Sp1* is a well-known transcription factor with pro-oncogenic function in multiple tumors, and so we selected it for subsequent research.

*Sp1* binds to guanine/cytosine (GC)-rich motifs of many promoters and is involved in many cellular processes, including cell differentiation, growth, and apoptosis; immune responses; response to DNA damage; and chromatin remodeling. It is overexpressed in multiple tumors and is a negative prognostic factor for patient survival. For example, Kim et al. studied the expression profile of 203 TNBC patients during adjuvant chemotherapy and found that an increase in *Sp1* expression was associated with poor prognosis. *Sp1* expression in a multivariate Cox regression model was an effective indicator for predicting the long-term prognoses of TNBC patients treated with doxorubicin [28]. In addition, a large number of studies have shown that *Sp1*-regulated genes are related to pro-oncogenic activity [35]. For example, the extracellular signal-regulated kinase (*ERK*)/*Sp1* signaling pathways mediate the *TGF-β*-induced *EGFR* upregulation, resulting in the promotion of BC cell migration and invasion [29]. Signal transducer and activator of transcription 3 (*STAT3*) and *Sp1* cooperate to induce high expression of the small GTPase Ras homolog family member U (*RhoU*) and enhanced BC cell migration [30]. In the present study, we found *Sp1* to be a target gene of miR-2110 and positively correlated with AFAP1-AS1 expression. Downregulation of *Sp1* suppressed the proliferation, migration, and invasion of MDA–MB-231 and MDA–MB-468 cells as well as tumor growth in vivo. In addition, AFAP1-AS1 was shown to competitively bind to miR-2110 to reduce the latter’s inhibitory effect on *Sp1*, resulting in the promotion of TNBC progression. All of the above findings indicated that AFAP1-AS1 served as a ceRNA to contribute to TNBC progression through the miR-2110/*Sp1* axis.

Some studies have shown that *Sp1*, as a sequence-specific DNA binding protein, could initiate the transcription of many cellular genes (including lncRNAs) and participate in various biological processes such as cell proliferation, differentiation, and tumor formation. For example, in retinal malformation cells, *Sp1* directly bound to the lncRNA PANDAR promoter region and promoted its transcription to regulate apoptosis caused by the B-cell lymphoma 2 (*Bcl-2*)/*caspase-3* pathway [36]. *Sp1* can bind lncRNA sprouty receptor tyrosine kinase signaling antagonist 4–intronic transcript 1 (*SPRY4*-*IT1*) promoter and activate its transcription, thereby playing a carcinogenic role in cholangiocarcinoma [37]. Of note, JASPAR platform (the open-access database of transcription factor binding profiles) predicted 18 *Sp1* binding sites in the AFAP1-AS1 promoter region, indicating that *Sp1* might bind to the AFAP1-AS1 promoter region to regulate the expression of AFAP1-AS1. Therefore, we presumed that AFAP1-AS1 acted as a miR-2110 sponge, reducing the inhibitory effect of miR-2110 on *Sp1*; in turn, the elevated *Sp1* bound to the AFAP1-AS1 promoter region and activated its transcription, forming a positive feedback system. Certainly, these hypotheses need further exploration and discovery in follow-up research.

In summary, we detected an lncRNA (AFAP1-AS1) that was overexpressed in TNBC tissues, and that upregulated *Sp1* by sponging miR-2110. Meanwhile, the AFAP1-AS1/miR-2110/*Sp1* axis modulated the proliferation, migration, and invasion of TNBC cells and affected tumorigenesis in mice. Our results not only elucidated the potential mechanism by which lncRNAs regulated the TNBC progression but also suggested that the AFAP1-AS1/miR-2110/*Sp1* axis could be a potential target in TNBC.

## Materials and methods

### Tissue collection

Fresh-frozen TNBC tissues and corresponding non-tumorous breast samples were obtained from Chinese patients at Peking Union Medical College Hospital (PUMCH, Beijing, China) during the period January–April 2021. No local or systemic treatment was conducted in these patients before the surgery. The study was approved by the Research Ethics Committee of PUMCH and informed consent was obtained from all patients.

### Cell culture

MCF-10A (iCell-h131, iCell Biotechnology Co. Ltd., Shanghai, China) was cultured in specific MCF-10A cell medium (iCell-h131-001b, iCell Biotechnology Co. Ltd., Shanghai, China). BT-549 (iCell-h029, iCell Biotechnology Co. Ltd., Shanghai, China) was cultured in the RPMI-1640 medium with 10% fetal bovine serum. MDA–MB-231 (ATCC® HTB-26™, The Chinese Academy of Sciences, Beijing, China) and MDA–MB-468 (BNCC339862, Bnbio, Beijing, China) cells were cultured in L15 medium containing 20% fetal bovine serum at 37 °C and 100% air constant temperature incubator. When cells grew to 80% confluence, the original medium was discarded and the cells were digested using 1 ml of 0.25% trypsin. After the adherent cells become round, we stopped the digestion with 1 ml medium and centrifuged the cells in a low-speed centrifuge at 1000 r/min for 3 min. The supernatant was aspirated, and an appropriate amount of culture medium was added to the cell pellet. Cells were passaged at the ratio of 1:4 to 1:3 every 2–3 days. We observed the morphological changes under an inverted microscope (Leica Microsystems, Shanghai, China).

### Plasmid construction

The shlncRNA–AFAP1-AS1 plasmid of lncRNA–AFAP1-AS1 was constructed to the pcDNA3.1-EGFP vector. Target sequences (shR–AFAP1-AS1-top: 5′- GATCCGTTCTGGGCTTCAATTTACAAGCAGTCAGCTCGAGCTGACTGCTTGTAAATTGAAGCCCAGAACTTTTTGA-3′; shR–AFAP1-AS1-bot: 5′- AGCTTCAAAAAGTTCTGGGCTTCAATTTACAAGCAGTCAGCTCGAGCTGAC TGCTTGTAAATTGAAGCCCAGAACG-3′) were inserted between the restriction sites of NheI (GCTAGC) and AgeI (ACCGGT).

The sh*Sp1* plasmid of *Sp1* was also constructed by the pcDNA3.1-EGFP vector. The target sequences (5′-GCTAGCGCTGGTGGTGATGGAATACATCTCGAGATGTATTCCATCACCACCAGCTTTTTGAATTC-3′) were inserted between the restriction sites of NheI (GCTAGC) and EcoRI (GAATTC).

### Cell transfection

Cells in the logarithmic-growth phase were used for subsequent experiments. The pcDNA3.1-NC plasmid and shlncRNA–AFAP1-AS1 plasmid were transiently transfected with Lipofectamine 3000 (L3000015, Thermo Fisher Scientific, Shanghai, China) according to the manufacturer’s instructions. Cells were seeded in a 12-well plate the day before transfection and cultured overnight. We transfected 1 μg plasmid per well when cells reaching 60–80% density. After transfection, cells were incubated at 37 °C for 72 h for subsequent experiments.

### RT-qPCR

Total RNA was extracted using TRIzol reagent (Invitrogen Corp., Carlsbad, CA, USA) and the RNA quantity and density were verified by a Nanodrop 2000 spectrophotometer. PrimeScript^TM^ RT Reagent Kit (RR037A, Takara, China) was used to reverse transcription. RT-qPCR was performed using the SYBR Premix Ex Taq^TM^ (RR820A, Takara, China) according to the manufacturer’s instructions. The assays were operated in triplicate and relative gene expression was determined by using the 2^−ΔΔCt^ method.

For mRNA RT-qPCR, we used 2 μg total RNA and random hexamers for the mRNA RT reaction. The lncRNA–AFAP1-AS1 primers 5′-AATGGTGGTAGGAGGGAGGA-3′ (sense) and 5′-CACACAGGGGAATGAAGAGG-3′ (antisense); *GAPDH* primers 5′- ATGACATCAAGAAGGTGGTGAAGCAGG-3′ (sense) and 5′-GCGTCAAAGGTGGAGGAGTGGGT-3′ (antisense); and *Sp1* primers 5′-TTGCTGCTATGCCAAACCTA-3′ (sense) and 5′-CCTGAGAGCTGGGAGTCAAG-3′ (antisense) were used in the mRNA qPCR reaction.

For miRNA RT-qPCR, we used 0.5 μg total RNA and miR-2110 RT Primer (or U6 RT Primer, Table S1) for miRNA RT reaction. The primers 5′-TGCGGTTGGGGAAACGGCCGCTG-3′ (miR-2110, forward), 5′-CCAGTGCAGGGTCCGAGGT-3′ (miR-2110, reverse); 5′-GCTCGCTTCGGCAGCACA-3′ (U6, forward) and 5′-AACGCTTCACGAATTTGCGTG-3′ (U6, reverse) were used in the miRNA qPCR reaction.

### CCK-8 and colony formation

For CCK-8 assay, we inoculated 5 × 10^3^ cells into each well of a 96-well plate after 72 h transfection. At each time point (0, 24, 48 and 72 h), we added 10 μL CCK-8 solution (CK04, Dojindo, China) to the wells. After 4 h of incubation, we determined the absorbance of each individual well at 450 nm and drew the growth curve with the obtained data.

For colony formation assay, cells were digested into a single-cell suspension with 500 cells seeded in each culture dish (6 cm). We added the appropriate complete medium to each dish and refreshed the culture medium every 3 days. Cells were then washed twice with PBS, fixed with 4% paraformaldehyde, and stained with 1 mL Giemsa stain for 15 min. We manually counted colonies and averaged the number from the duplicate wells. Clone formation rate (%) = (number of cell clones/total number of cells added) × 100%.

### Wound healing migration and transwell invasion assays

For wound healing migration assay, we mechanically disrupted cell monolayers using a sterile 10 μL micropipette tip to generate a linear wound, and then we washed off the cells with PBS. After that, the culture medium was added. The 24-well plates were taken out of the incubator at 0, 24 and 48 h for micrograph. We observed the migration distance of cells, calculated their migration rates at different time points and drew the column diagram. Migration rate (%) = (scratch area of 0 h−scratch area of *N* h)/(scratch area of 0 h) ×100%.

For Transwell invasion assays, cells were incubated in 24-well Transwell plates (8 μm pore size, Corning, NY, USA). We plated 1 × 10^5^ cells suspended in serum-free medium in the upper chambers with Matrigel (BD Biosciences, Franklin Lakes, NJ, USA), and added 600 μL complete medium to the lower chamber. After incubation for a suitable amount of time(24 h for MDA–MB-231 and 48 h for MDA–MB-468), the cells were fixed in 4% iced paraformaldehyde for 30 min, stained by Giemsa stain for 15 min and counted under the inverted microscope (Leica Microsystems, Shanghai, China).

### Luciferase reporter assay

Vectors of luciferase reporters were synthesized to pmirGLO vector to construct wild-type and mutation plasmids. Cells were inoculated into a 24-well plate and were co-transfected with miRNA mimics or the NC plus the luciferase reporter vector as well as lipofectamine 3000 reagent. After 48 h, the dual-luciferase reporter was detected by Dual-Luciferase® Reporter Assay System (E1910, Promega Corp., Fitchburg, WI, USA), and relative luciferase activity was normalized to Renilla luciferase activity. The sequence of miRNA-NC mimics was 5′-UCACAACCUCCUAGAAAGAGUAGA-3′, and the sequence of hsa-miR-2110 mimics was 5′-UUGGGGAAACGGCCGCUGAG UG-3′.

### Western blot assays

For total protein extraction, cell lysates were obtained using RIPA lysis. A total of 10 μL protein was injected into a 10% SDS-PAGE gel and transferred to PVDF membranes at 4 °C, 200 mA for 1 h. After blocking with blocking solution, the membranes were incubated with primary antibody overnight at 4 °C. After being washed in PBS, membranes were then exposed to the secondary antibody (HRP-Goat anti rabbit/mouse IgG antibody) for 1 h. We incubated bands using an electrochemiluminescence (ECL) kit (P1052; Applygen Technologies Inc. Beijing, China) and analyzed with an imaging system. The primary antibodies CLDN4 (16195-1-AP), *Sp1* (21962-1-AP), RALYL (17179-1-AP), RHBDD1 (20869-1-AP), ZNF703 (21075-1-AP), p38 (140641-1-AP), JNK (66210-1-Ig), p-JNK (80024-1-RR), MDM2 (19058-1-AP), VEGF (19003-1-AP), survivin (10508-1-AP), GAPDH (60004-1-Ig) were all purchased from Proteintech (Chicago, IL, USA), and the primary antibody p-p38 (YP0338) was purchased from Immunoway Biotechnology Company (Plano, TX, USA).

### In vivo assays

We purchased 72 BALB/C specific-pathogen-free (SPF) nude mice, 1–5-weeks old, from SPF (Beijing) Biotechnology Co., Ltd. (Beijing, China). All mice were female (not pregnant), healthy and mature, with an average weight of 18 ± 2 g. They were raised in the barrier system of the Laboratory Animal Center of the Institute of Radiation Medicine, Chinese Academy of Medical Sciences. All feed, water, air, bedding, and various supplies that enter the barrier system must be sterilized by high temperature and high pressure; all people and animals entering the laboratory must undergo strict microbial control.

After 1 week of adaptive growth, they were randomly divided into 12 groups (*n* = 6 per group). We subcutaneously inoculated 0.1 ml of 10^8^/ml cells diluted with PBS into mouse axillae. The mice’s mental state, activity, responsiveness, diet, and appearance of the subcutaneous vaccination area were observed every 2 days. We measured tumor size every week using a Vernier caliper and calculated tumor volume (*V*) as *V* (cm^3^) = 1/2AB^2^, with A and B representing the largest and smallest diameters, respectively. With the tumor volume plotted along the *Y*-axis and weeks of growth plotted along the *X*-axis, we drew the tumor growth curves of each group. Animals were euthanized 30 days after inoculation, and the tumors were removed, weighed, and then placed in 4% paraformaldehyde for fixation.

### Data analysis

The data obtained were expressed as mean ± standard deviation (SD) or standard error (SE). We performed one-way analysis of variance (ANOVA) and Student’s *t* test to analyze intergroup differences. Spearman’s correlation coefficient was used to measure the linear relationships between distance variables. *P* < 0.05 indicated significant differences.

## Supplementary information

Supplemental Material
