# Supplementary material for: Long noncoding RNA AFAP1-AS1 promotes tumor progression and invasion by regulating the miR-2110/Sp1 axis in triple-negative breast cancer
Source: Cell Death Dis. 2021 Jun 18;12(7):627. doi: 10.1038/s41419-021-03917-z (PMC8213778; doi:10.1038/s41419-021-03917-z)
Supplement: Supplementary file 1 — Supplemental Material [file 41419_2021_3917_MOESM1_ESM.pdf]

Table S1. Primers or sequences used in the study.

shlncRNA-AFAP1-AS1 plasmid	shR-AFAP1-AS1-top	5'-GATCCGTTCTGGGCTTCAATTTACAAGCAGTCAGCTCGAGCTGACTGCTTGTAAATTGAAGCCCAGAACTTTTTGA-3'
	shR-AFAP1-AS1-bot	5'-AGCTTCAAAAAGTTCTGGGCTTCAATTTACAAGCAGTCAGCTCGAGCTGACTGCTTGTAAATTGAAGCCCAGAACG-3'
sh*Sp1* plasmid	Sh*Sp1* target sequence	5'-GCTAGCGCTGGTGGTGATGGAATACATCTCGAGATGTATTCCATCACCACCAGCTTTTTGAATTC-3'
RT Primer	miR-2110	5'-GTCGTATCCAGTGCAGGGTCCGAGGTGCACTGGATACGACCACTCAG-3'
	U6	5'-AACGCTTCACGAATTTGCGTG-3'
qPCR Primer	LncRNA-AFAP1-AS1	5'-AATGGTGGTAGGAGGGAGGA-3' (sense)<br>5'-CACACAGGGGAATGAAGAGG-3' (antisense)
	GAPDH	5'-ATGACATCAAGAAGGTGGTGAAGCAGG-3' (sense)<br>5'-GCGTCAAAGGTGGAGGAGTGGGT-3'(antisense)
	miR-2110	5'-TGCGGTTGGGGAAACGGCCGCTG-3' (forward)<br>5'-CCAGTGCAGGGTCCGAGGT-3' (reverse)
	U6	5'-GCTCGCTTCGGCAGCACA-3' (forward)<br>5'-AACGCTTCACGAATTTGCGTG-3' (reverse)
	Sp1	5'-TTGCTGCTATGCCAAACCTA-3' (sense)<br>5'-CCTGAGAGCTGGGAGTCAAG-3' (antisense)
mimics	miRNA-NC mimics	5’-UCACAACCUCCUAGAAAGAGUAGA-3’<br>3’-AGUGUUGGAGGAUCUUUCUCAUCU-5’
	hsa-miR-2110 mimics	5’-UUGGGGAAACGGCCGCUGAGUG-3’<br>3’-AACCCCUUUGCCGGCGACUCAC-5’
Inhibitor	miRNA inhibitor control	5’-UCUACUCUUUCUAGGAGGUUGUGA-3’
	miR-2110 inhibitor	5’-CACUCAGCGGCCGUUUCCCCAA-3’

Table S2. Clinicopathological features of the eight patients.

Patient No.	Age (years)	Tumor size (cm)	Number of positive lymph nodes
1	36	2.20	1
2	47	3.10	0
3	55	1.10	0
4	64	0.90	0
5	49	2.10	0
6	41	2.00	0
7	57	1.60	0
8	70	1.50	2

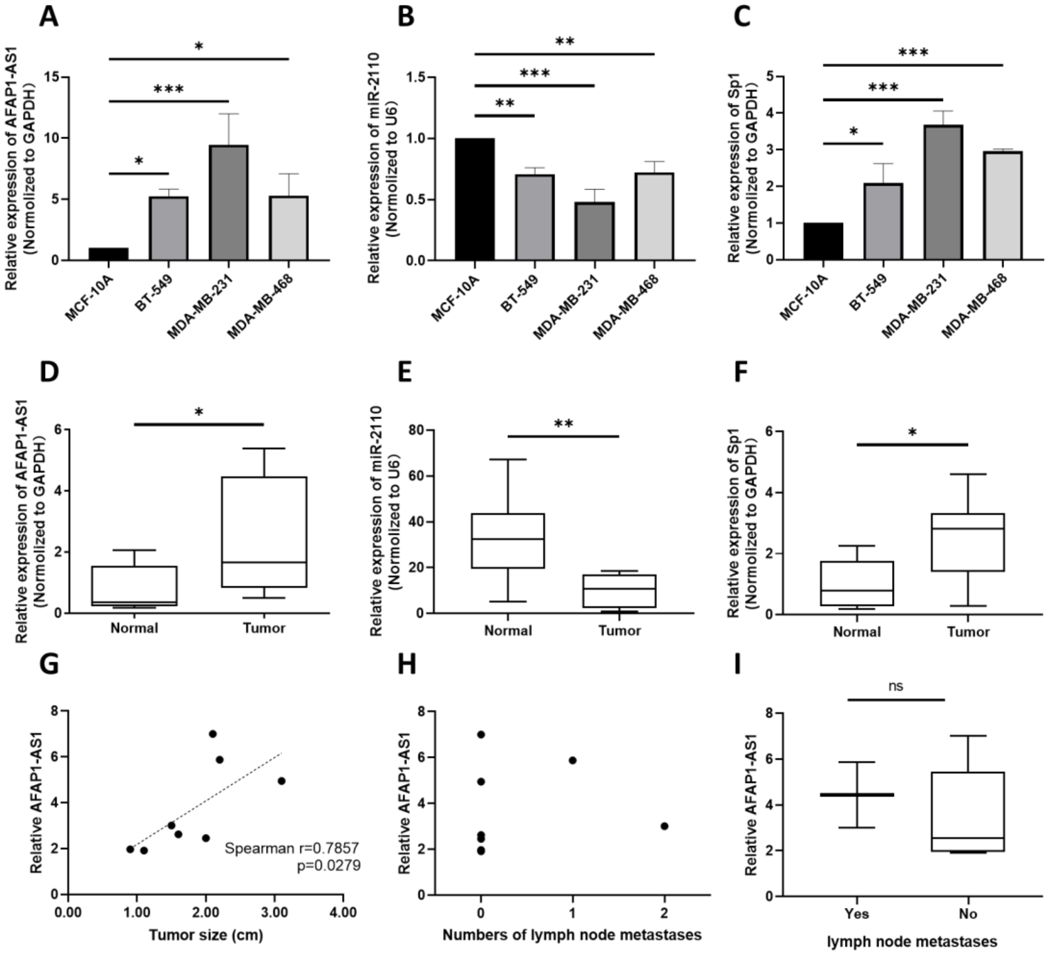

Figure S1. Relative mRNA expression of (A) AFAP1-AS1, (B) miR-2110 and (C) *Sp1* in TNBC cells (*i.e.*, BT-549, MDA–MB-231, and MDA–MB-468) comparing with normal human breast epithelial cell line MCF-10A. Relative mRNA expression of (D) AFAP1-AS1, (E) miR-2110 and (F) *Sp1* in TNBC tumor tissues and normal tissues. Unpaired student's *t*-tests were used for the statistical analyses. The correlation of tumor size with clinicopathological features, like (G) tumor size (Spearman correlation coefficient was used to measure the linear relationship between distance variables), and (H–I) numbers of lymph node metastases (Unpaired student's *t*-tests were used). *$P < 0.05$; **$P < 0.01$; ***$P < 0.005$; ns, not significant.

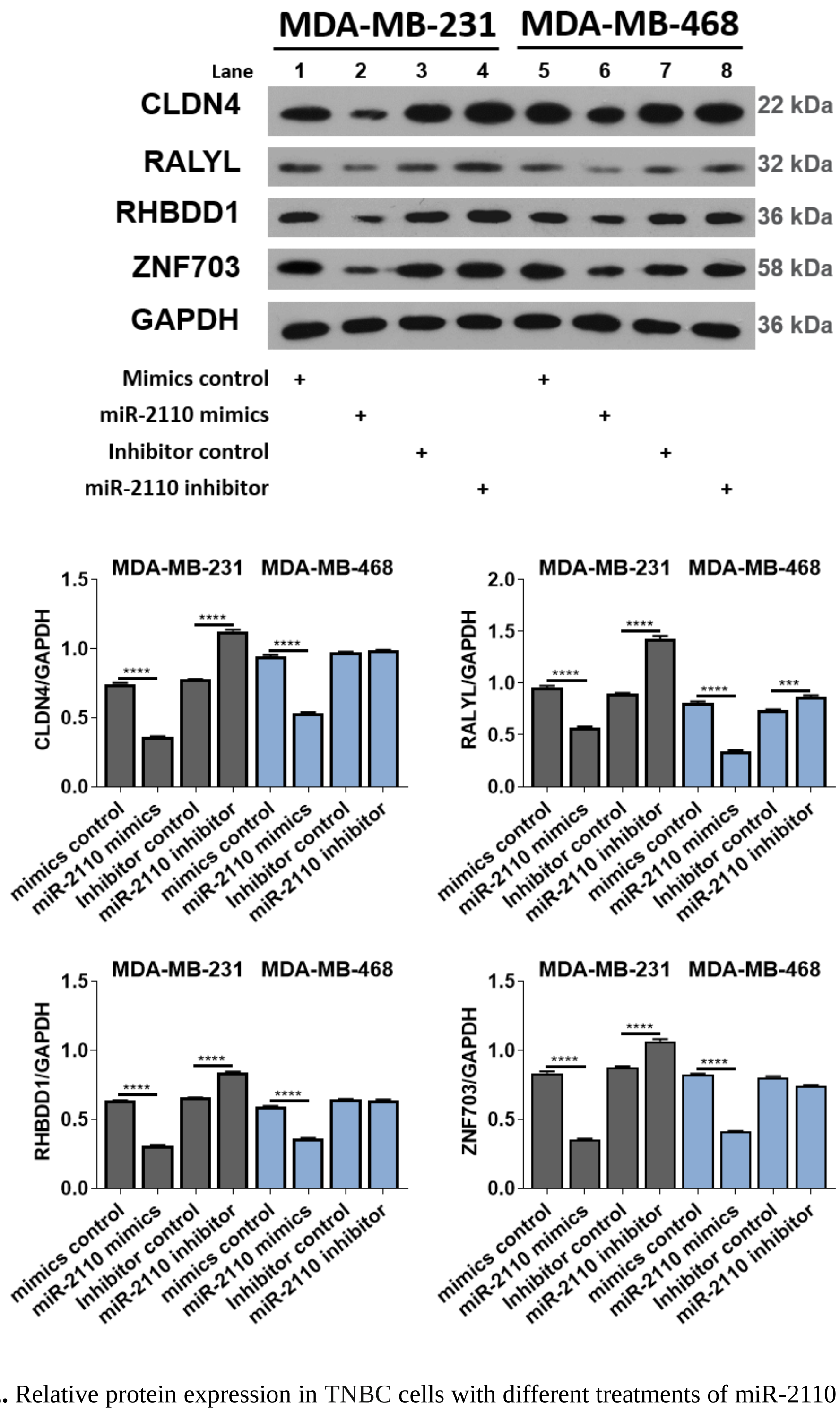

Figure S2. Relative protein expression in TNBC cells with different treatments of miR-2110 mimics or inhibitor. Unpaired student's *t*-test and one-way ANOVA test were used for the statistical analyses. **P* < 0.05; ***P* < 0.01; ****P* < 0.005;**** *P* < 0.001; ns, not significant.

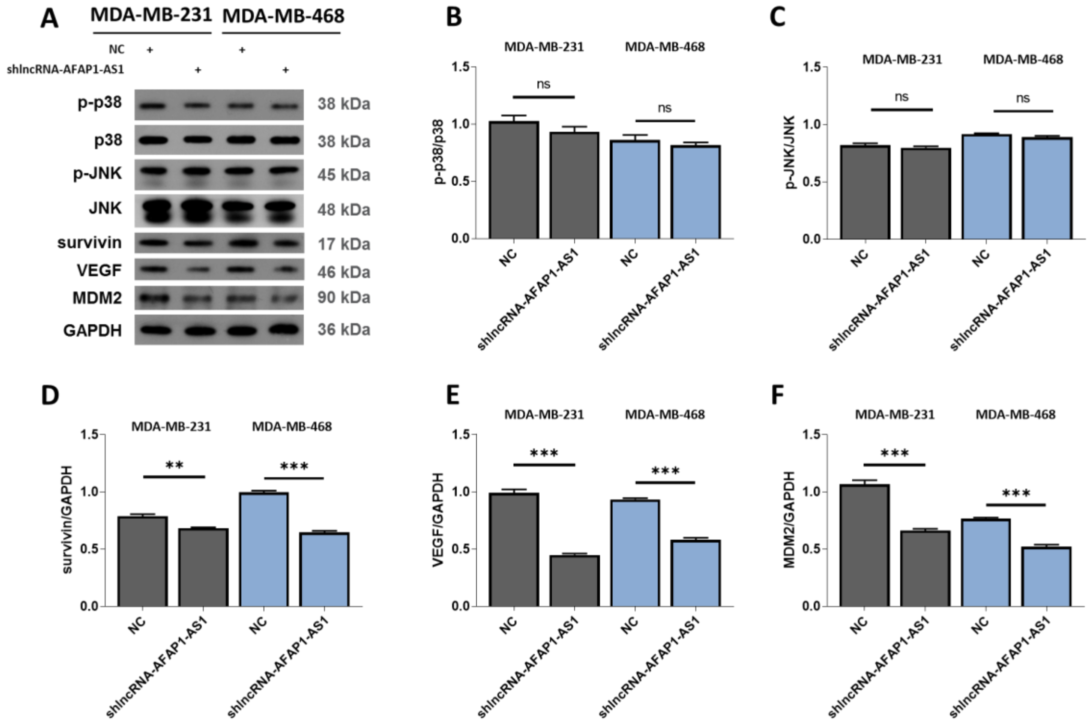

Figure S3. Effects of AFAP1-AS1 on *Sp1* related pathway proteins. (A) WB assay results. The reduction in AFAP1-AS1 did not affect protein expression of (B) *p-p38* or (C) *p-JNK*, indicating that AFAP1-AS1 did not regulate the upstream of *Sp1*-MAPK signaling pathway. Downregulation of AFAP1-AS1 modulated the downstream proteins (D) *survivin*, (E) *VEGF* and (F) *MDM2*. Unpaired student's *t*-test and one-way ANOVA test were used for the statistical analyses. *$P < 0.05$; **$P < 0.01$; ***$P < 0.005$; ns, not significant.

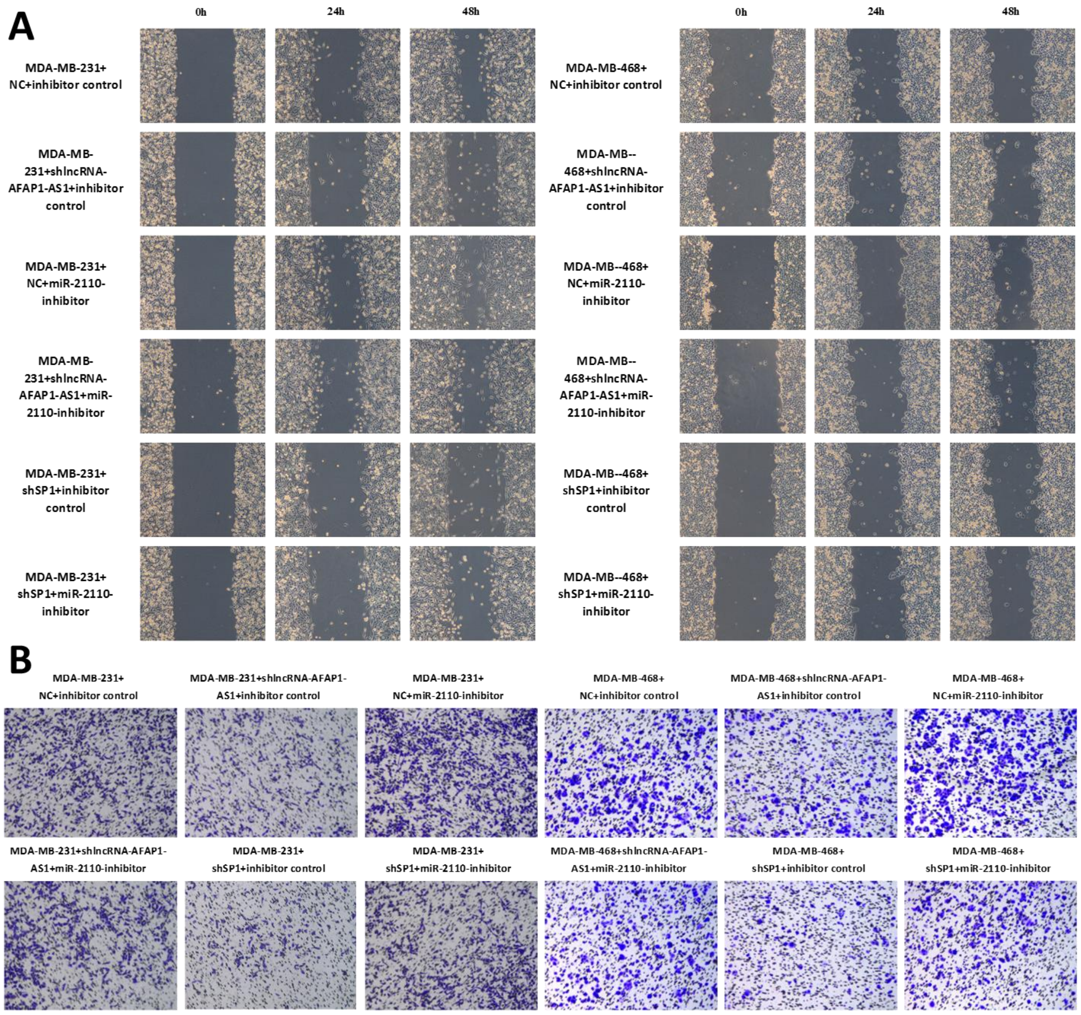

Figure S4. (A) Wound healing migration and (B) Transwell invasion assays performed in MDA–MB-231 and MDA–MB-468 cells. Quantitative results are shown in Figure 4D and 4E.

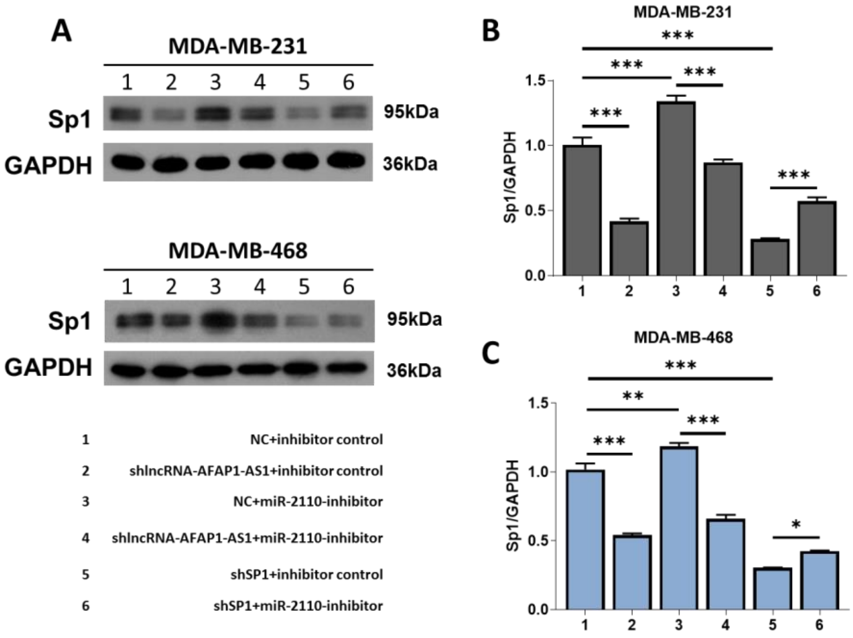

Figure S5. (A) Protein levels of *Sp1* in mice tumors were verified. *Sp1* protein levels in (B) MDA–MB-231 and (C) MDA–MB-468 injection group. Unpaired student's *t*-test and one-way ANOVA test were used for the statistical analyses. *P < 0.05; **P < 0.01; ***P < 0.005; ns, not significant.
